# The novel protein C3orf43 accelerates hepatocyte proliferation

**DOI:** 10.1186/s11658-017-0051-3

**Published:** 2017-09-16

**Authors:** Chunyan Zhang, Cuifang Chang, Deming Li, Fuchun Zhang, Cunshuan Xu

**Affiliations:** 1 Xinjiang Key Laboratory of Biological Resources and Genetic Engineering, College of Life Science and Technology, Xinjiang University, Urumqi, 830046 China; 2 State Key Laboratory Cultivation Base for Cell Differentiation Regulation and Henan Engineering Laboratory for Bioengineering and Drug Development, College of Life Science, Henan Normal University, Xinxiang, Henan 453007 China

**Keywords:** Novel protein, C3orf43, Cell proliferation, siRNA, Overexpression vector

## Abstract

**Background:**

Our previous study found that single-pass membrane protein with coiled-coil domains 1 (C3orf43; XM_006248472.3) was significantly upregulated in the proliferative phase during liver regeneration. This indicates that C3orf43 plays a vital role in liver cell proliferation. However, its physiological functions remains unclear.

**Methods:**

The expressions of C3orf43 in BRL-3A cells transfected with C3orf43-siRNA (C3-siRNA) or overexpressing the vector plasmid pCDH-C3orf43 (pCDH-C3) were measured via RT-qPCR and western blot. Cell growth and proliferation were determined using MTT and flow cytometry. Cell proliferation-related gene expression was measured using RT-qPCR and western blot.

**Results:**

It was found that upregulation of C3orf43 by pCDH-C3 promoted hepatocyte proliferation, and inhibition of C3orf43 by C3-siRNA led to the reduction of cell proliferation. The results of qRT-PCR and western blot assay showed that the C3-siRNA group downregulated the expression of cell proliferation-related genes like JUN, MYC, CCND1 and CCNA2, and the pCDH-C3 group upregulated the expression of those genes.

**Conclusion:**

These findings reveal that C3orf43 may contribute to hepatocyte proliferation and may have the potential to promote liver repair and regeneration.

## Introduction

The liver has an extraordinary ability to regenerate. After injury or partial hepatectomy, the remaining liver tissue proves capable of quickly regenerating to reach its original size and weight, with a restoration of tissue structure and organ function [1, 2]. Liver regeneration involves multiple processes, including initiation, proliferation and termination [3, 4].

The regenerative capacity of the liver is essential for the success of many treatments focused on this organ, including transplants and the elimination of liver tumors. Understanding the molecular mechanisms underlying tissue regeneration and developing a novel strategy to gain insight into the liver regeneration process is crucial to further development of successful treatments [5].

Our previous proteomics research using two-dimensional gel electrophoresis (2D) and mass spectrometry (MS) found that C3orf43 was remarkably upregulated in the proliferation stage of liver regeneration. Western blot was used to verify this C3orf43 upregulation, with analyses run 12, 24, 30, 36 and 72 h after partial hepatectomy. We hypothesized that C3orf43 may be associated with liver regeneration.

The official full name of C3orf43 is single-pass membrane protein with coiled-coil domains 1 (SMCO1). Some studies annotated it, but did not study its function [6, 7]. Its full length is 2653 bp, and it contains three exons and two introns. The exons are located at 1–183, 1593–1742 and 2139–2653 bp. Its mRNA has a full length of 886 bp and encodes 214 amino acids. The sequences of three homologous genes of rat, mouse and human have similar structures and almost the same CDS length. The amino acid sequence similarity for rat, mouse and human reached 91.27% (assessed using Cluster X software). Three homology proteins share a conserved domain of DUF4547, typically 144–206 amino acids long, with unknown function. This domain family was discovered in eukaryotes.

To elucidate the role of C3orf43 in hepatocytes, we focused on the pro-proliferative function of C3orf43 in rat liver cell line BRL-3A. Based on our data, we hypothesize that C3orf43 promotes proliferation in rat liver cell line BRL-3A by modulating cell cycle transition and the expression of the key genes JUN, MYC, CCND1 and CCNA2.

## Materials and methods

### Preparation of rat liver regeneration model

Adult male Sprague–Dawley (SD) rats, weighing 230 ± 20 g, were obtained from the Animal Center of Henan Normal University. Their conditions were approved by the Animal Care and Use Committee of the Henan Normal University (permit number SYXK2008–0105). The rats were housed in a room at 21 ± 2 °C with a relative humidity 60 ± 10% and illumination for 12 h/day, from 8:00–20:00. They had free access to water and food. The 76 rats were randomly divided into 19 groups of 4, including 9 partial hepatectomy (PH) groups, 9 sham-operated (SO) groups and one normal control (NC) group. The rats in the PH group were subjected to two-thirds hepatectomy following the method of Higgins and Anderson (1931). The SO group underwent the same procedure as the PH group without liver removal. After the operation, the abdominal cavities of the rats were opened at 0, 2, 6, 12, 24, 30, 36, 72, 120 and 168 h to collect liver tissue. The whole handling procedures was carried out in accordance with the current animal protection laws of China.

### Cell culture

The human embryo kidney cell line 293 T and rat liver cell line BRL-3A were obtained from the cell bank of the School of Basic Medicine of Peking Union Medical College, China. Cells were maintained at 37 °C in a humidified incubator with 5% CO_2_, and cultured in Dulbecco’s modified Eagle’s medium (DMEM, Life Technologies) supplemented with 100 U/mL penicillin and streptomycin and 10% fetal bovine serum (Gibco).

### Two-dimensional gel electrophoresis (2D) and mass spectrometry (MS)

2D/MS was performed according to the previously described method [8], with 2D done as described in the operating manual for the instrument (GE Healthcare) and three times for each sample, and protein identification conducted via matrix-assisted laser desorption ionization time-of-flight mass spectrometry (MALDITOF/MS) using a Bruker Daltonics Autoflex III.

### The design and synthesis of siRNA targeting C3orf43

BRL-3A cells were transfected with siRNA targeting C3orf43 (Table 1). A siRNA oligonucleotide sequence consisting of a scrambled sequence that does not recognize any mammalian gene was used as the negative control. Cells were transfected with the indicated siRNA (50 nM) using Lipofectamine RNAiMAX (Invitrogen) according to the manufacturer’s instructions. The expression change of C3orf43 was determined via RT-PCR 48 h after transfection.

**Table 1:** The sequence of C3orf43 siRNAs

siRNA	Target sequence	Sequence
*C3orf43* siR1	GCTCCAAGCTCTAGACACA	5’GCUCCAAGCUCUAGACACA dTdT3’
		3’dTdTCGAGGUUCGAGAUCUGUGU5’
*C3orf43* siR2	CCTCAATGGAACTGAATAT	5’CCUCAAUGGAACUGAAUAUdTdT3’
		3’dTdTGGAGUUACCUUGACUUAUA5’
*C3orf43* siR3	CCTCTGTTCTTAGAAGAAA	5’CCUCUGUUCUUAGAAGAAAdTdT3’
		3’dTdTGGAGACAAGAAUCUUCUUU5’

### Plasmid constructs and lentivirus production

Coding sequences of rat C3orf43 (XM_006248472.3) were synthesized and inserted into the multiple cloning site (MCS) of the lentiviral vector pCDH-CMV-MCS-EF1-copGFP (pCDH) by Generay Biotech. Briefly, vector particles were produced in HEK293T cells via transient co-transfection involving a three-plasmid expression system. Viral packaging was processed according Dai et al. [9] and Ding et al. [10]. Then, the concentrated virus particles were suspended in PBS and stored at −80 °C for the subsequent experiments.

### Transduction of BRL-3A

Transduction was performed in 24-well plates. BRL-3A were seeded at 1 × 10^5^ cells per well and 16 h later, the cells were transduced with 2 × 10^5^ TU virus particles of C3orf43 for 8 h. The viral infection was serially repeated 2–3 times. Three days after the last round of transduction, the efficiency was measured by detecting the green fluorescent protein (GFP) using a fluorescence microscope. After 1 or 2 weeks, the transduced cells in clusters were partially digested and seeded into new dishes to continue their culture.

### RNA isolation and quantitative RT-PCR analysis

Total cellular RNA was extracted using Trizol (Invitrogen Corporation) according to the manufacturer’s instructions. The integrity of the RNA was determined using denatured agarose gel electrophoresis (70 v, 20 min). RNA purity was analyzed using a spectrophotometer at with absorbance values of 260 nm and 280 nm (A260/280). Qualified RNA (2 μg) was used to synthesize the first strand of cDNA following the reverse transcription kit (Promega). The cDNA was used for PCR amplification and GAPDH was used as a reference gene. The sequences of the primers used in this study, including cell proliferation-related genes, are listed in Table 2.

**Table 2:** The primer sequences used in the RT-PCR

Genes	Forward primer	Reverse primer
C3orf43	5′- GTCTTCCGACTTTAGCCTCTG-3’	5′- GCATCCGTCTTACATTAGCG-3’
Ccna2	5′-CTTTTAGTGCCGCTGTCTCTTT-3’	5′-GCCCGCATACTGTTAGTGATGT-3’
*Myc*	5′-ACCCAACATCAGCGGTCG-3’	5′-CGTGACTGTCGGGTTTTCCA-3’
*Ccnd1*	5′-AAAATGCCAGAGGCGGATGA-3’	5′-GAAAGTGCGTTGTGCGGTAG-3’
*Jun*	5′-TGCAAAGATGGAAACGACCTT-3’	5′-GCCGTAGGCGCCACTCT-3’

### MTT assay

The MTT assay was used to measure the cell viability of cells. Briefly, after cells with 0.02 ml of 5 mg/ml MTT solution (Sigma) were incubated for 4 h, then the medium was replaced and with 0.15 ml of dimethylsulfoxide (DMSO; Sigma) was added and the plates were gently shaken for 10 min at room temperature. The absorbance was measured at 490 nm using a Biotek ELx800 reader. All the treatments were done in three duplicates, and three independent experiments were performed.

### Cell cycle analysis

The cell cycle was analyzed using flow cytometry. 1 × 10^5^ cells/well were seeded into 24-well plates and transfected with C3orf43-siRNA (C3-siRNA) or negative control, or with pCDH-C3orf43 (pCDH-C3) or empty vector (pCDH). Cells were harvested 48 h post-transfection. After harvest, cells were washed in cold PBS and then fixed in 70% alcohol at −20 °C for at least 12 h. The fixed cells were washed 3 times in cold PBS and incubated in 1 ml of PBS solution with 50 μg of propidium iodide (PI, Sigma) and 100 μg of RNase A (Sigma) for 30 min at 37 °C. The samples were then analyzed for DNA content using FACSCan.

### Western blot analysis

Cells were lysed on ice with RIPA buffer (50 mM Tris, 150 mM NaCl, 1% Triton X-100, 1% sodium deoxycholate and 0.1% SDS) containing proteinase inhibitors (1 mM phenylmethylsulfonyl fluoride, 2 μg/ml aprotinin and 2 μg/ml leupeptin). Cell lysates were boiled in SDS sample buffer for 5 min. An equal amount of protein per sample was separated via SDS-PAGE and transferred to nitrocellulose membranes (GE Healthcare). The membranes were first blocked with 5% non-fat milk in Tris-buffered saline (TBS) containing 0.1% Tween-20 (TBS-T). Then the membrane was incubated with rabbit anti-JUN (Boster, 1:1000), rabbit anti-MYC (Boster, 1:1000), rabbit anti-CCND1 (Bioss, 1:1000) and rabbit anti-CCNA2 (Boster, 1:1000) overnight at 4 °C. The membrane was further incubated with the horseradish peroxidase-conjugated secondary antibody goat anti-rabbit IgG (Sigma, 1:5000) after 3 washes with TBS-T at room temperature. Finally, the protein band was visualized with Amersham enhanced chemiluminescence (ECL) substrates (Western Lightning Plus-ECL). The band density was measured using ImageQuant TL software. b-actin (Sigma, 1:1000) served as an internal reference.

### Statistical analysis

All data were described as means ± SD of at least three independent experiments. Significance was assessed using one-way ANOVA following appropriate transformation to normalized data and equalized variance where necessary. Statistical analysis was performed using SPSS statistics 19.0 (SPSS Inc.). A *p* value less than 0.05 was considered significant and less than 0.01 was considered extremely significant.

## Results

### C3orf43 Content in regenerating rat liver

**Fig. 1:**
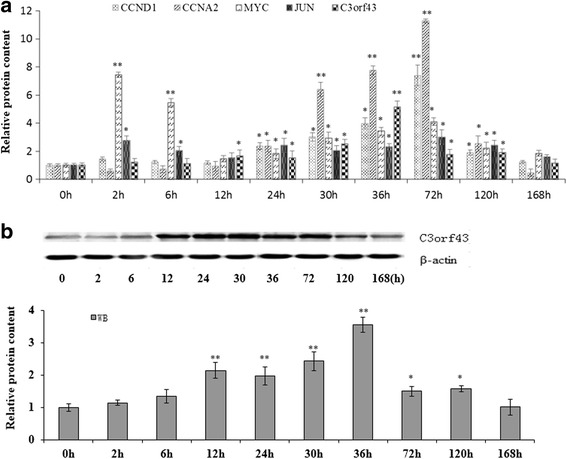
The changes in content of C3orf43, JUN, MYC, CCND1 and CCNA2 in the regenerating rat liver. **a** The content of C3orf43, JUN, MYC, CCND1 and CCNA2 at 0, 2, 6, 12, 24, 30, 36, 72, 120 and 168 h after partial hepatectomy in the regenerating rat liver was assessed by 2D/MS. **b** The content of C3orf43 at 0, 2, 6, 12, 24, 30, 36, 72, 120 and 168 h after partial hepatectomy in the regenerating rat liver was assessed by western blot. The experiments were performed three times with three replicates in each set. The data are shown as the means ± SD. Representative images were shown. *p < 0.05, **p < 0.01 difference with the levels at 0 h

2D/MS was used to assess the content of C3orf43 0, 2, 6, 12, 24, 30, 36, 72, 120 and 168 h after partial hepatectomy in the regenerating rat liver. We found that C3orf43 was remarkably upregulated at 12, 24, 30, 36 and 72 h, and that the proliferation-related genes, including JUN, MYC, CCND1 and CCNA2, were also upregulated in this stage of liver regeneration (Fig. 1a). Therefore, we hypothesized that C3orf43 was associated with cell proliferation. To verify the reliability of 2D/MS, western blot was employed. The results were consistent and confirmed the C3orf43 content changes at the 10 time points after partial hepatectomy (Fig. 1b).

### Virus preparation and identification of C3orf43 overexpression

**Fig. 2:**
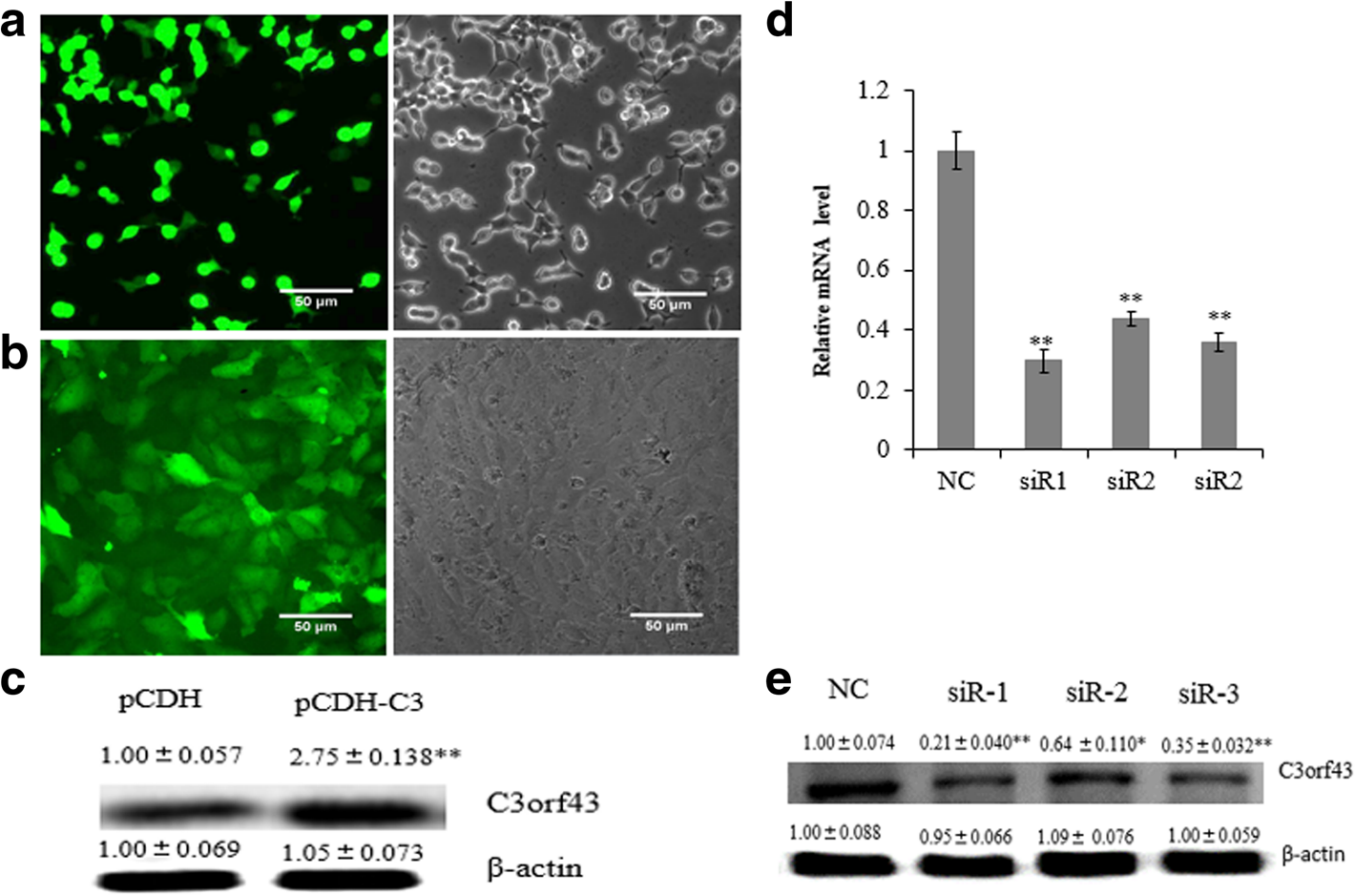
Preparation of virus and identification of C3orf43 overexpression and knockdown. **a** 293 T cells transfected with pCDH-C3 were observed with fluorescence microscopy. The bright field is to the left and the fluorescence image is to the right. **b** BRL-3A cells infected with lentiviral particles were observed with fluorescence microscopy. The bright field is to the left and the fluorescence image is to the right. **c** The C3orf43 content in the pCDH-C3 group was evaluated via western blot. **d** and **e** The expression of C3orf43 was determined using qRT-PCR and western blot. NC, siR1, siR2 and siR3 represent the cells treated with negative siRNA, siR1-C3orf43, siR2-C3orf43 or siR3-C3orf43, respectively. The experiments were performed three times with three replicates in each case. The data are shown as the means ± SD. Representative images are shown. *p < 0.05, **p < 0.01 difference relative to the control

The pCDH-C3 was transfected into 293 T cells. Around 90% of the cells exhibited high-intensity fluorescence 24 h after transfection, indicating successful viral packaging (Fig. 2a). After filtration, the concentrated virus titer was 1.5 × 108 TU/ml. This could be used for subsequent experiments.

About 95% of the lentivirus-infected BRL-3A cells exhibited high-intensity fluorescence 72 h after infection (Fig. 2b). Western blot analysis indicated that the C3orf43 content in pCDH-C3 group significantly increased compared with the empty vector pCDH group (pCDH; Fig. 2c; *p* < 0.01).

### The effect of siRNAs on C3orf43 expression

To verify the effect of siRNA on C3orf43 expression in BRL-3A cells, three siRNAs were designed based on the C3orf43 sequence, and qRT-PCR and western blot were used to determine the effects of different siRNAs on the expression of C3orf43. qRT-PCR analysis showed that C3orf43 expression in cells transfected with siR1 was knocked down to 30% compared to the negative control, whereas siR2 reduced the C3orf43 expression to 44% and siR3 to 36% (Fig. 2d). Western blot analysis indicated that C3orf43 content in cells transfected with siR1 was knocked down to 21% compared to the negative control, whereas siR2 reduced the C3orf43 expression to 64% and siR3 to 35% (Fig. 2e). Statistical analysis revealed that C3orf43 expression significantly declined in BRL-3A cells transfected with siR1, siR2 and siR3 (*p* < 0.01 vs. control) 48 h post-transfection. We used siR1 to perform the subsequent experiments.

### C3orf43 Promotes hepatocyte proliferation and cell growth

**Fig. 3:**
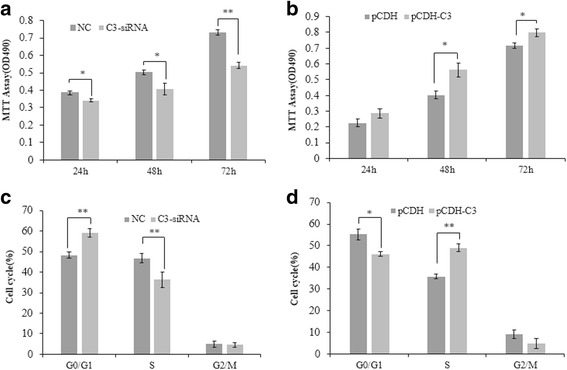
C3orf43 promotes hepatocyte proliferation and cell growth. **a** The cell viability was assessed using the MTT assay 24, 48 and 72 h after transfection with siRNA. **b** The cell viability was assessed using the MTT assay in cells with overexpression of pCDH-C3orf43. **c** Cell cycle distribution in the C3-siRNA and negative control (NC) groups, C3-siRNA vs. NC (S %: 36.31 ± 1.3% vs. 46.80 ± 1.1%, *p* < 0.01). **d** Cell cycle distribution in the pCDH-C3 and pCDH groups, pCDH-C3 vs. pCDH (S %: 49.25 ± 1.7% vs. 35.78 ± 1.2%, *p* < 0.01). The experiments were performed three times with three replicates in each case. The data are shown as the means ± SD. *p < 0.05, **p < 0.01 difference relative to the control

To determine the role of C3orf43 in the growth and proliferation of BRL-3A rat liver cells, MTT assay and cell cycle analysis were employed. As shown in Fig. 3a, C3-siRNA significantly suppressed BRL-3A cell growth at 24, 48 and 72 h, while pCDH-C3 promoted BRL-3A cell growth by MTT assay (Fig. 3b). In addition, cell cycle analysis by flow cytometry showed that the cells in C3-siRNA group exhibited a decrease in the cell population in S phase and a corresponding increase in G1 phase compared to the negative control group 48 h after transfection (Fig. 3c). The cells in the pCDH-C3 group exhibited an increase in the cell population in S phase and a corresponding decrease in G1 phase compared to the pCDH group. These results suggest that C3orf43 may be a promoter of the growth and proliferation of rat liver cells (Fig. 3d).

### The effect of C3orf43 on BRL-3A cell proliferation-related genes

**Fig. 4:**
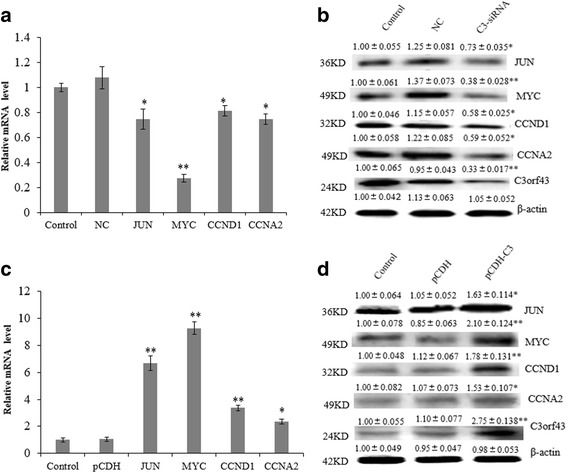
The effect of C3orf43 on BRL-3A cell proliferation-related genes. **a** The expressions of cell proliferation-related genes were assessed using qRT-PCR 48 h after transfection with siRNA. **b** The contents of cell proliferation-related genes were assessed using western blot 48 h after transfection with siRNA. **c** The expressions of cell proliferation-related genes were assessed using qRT-PCR in cells with overexpression of pCDH-C3orf43. **d** The content of cell proliferation related genes were assessed using western blot in cells with overexpression of pCDH-C3orf43. The experiments were performed three times. The data are shown as the means ± SD. Representative images are shown. *p < 0.05, **p < 0.01 difference relative to the control

To further determine the role of C3orf43 in the growth and proliferation of BRL-3A rat liver cells, qRT-PCR and western blot were used to detect the expression changes of proliferation-related genes. As shown in Fig. 4a and b, C3-siRNA significantly suppressed the expression of JUN, MYC, CCND1 and CCNA2 both at the mRNA and protein levels. pCDH-C3 increased the expression of JUN, MYC, CCND1 and CCNA2 both at mRNA and protein levels (Fig. 4c and d). The data indicate that C3orf43 promotes cell proliferation.

## Discussion

The liver is vulnerable to various injuries, including viruses, bacteria, the effects of alcohol and physical injury, but it has an enormous capacity for regeneration [4, 11, 12]. Liver regeneration is a complex process involving many factors and signaling pathways, including cytokines and growth factor networks [4]. Previous research has shown that the initiation of liver regeneration is driven by cytokine release [13].

TNF, NFκB and IL-6 are important mediators leading to the activation of STAT3 in hepatocytes. After partial hepatectomy, TNF binds to TNFR1, which induces NFκB activation and IL-6 production [14]. Then IL-6 interacts with IL-6R to activate STAT3 and ERK1/2 pathways [3, 15].

The EGFR ligand family and HGF are important growth factors during liver regeneration [16]. HGF and c-met, the gene for the HGF receptor, are essential for liver regeneration [17]. HGF/c-met signaling results in activation of ERK1/2 [18].

While these factors are known to be involved, it remains crucially significant to elaborate the underlying mechanism of liver regeneration after partial hepatectomy and to understand the cellular and molecular events that regulate hepatocyte proliferation. The liver’s regenerative potential is critical for the success of liver treatments, and a better understanding of these mechanisms and events would certainly benefit medical science.

To further the understanding of the molecular mechanism of liver regeneration, we performed proteomic studies of liver regeneration and found that C3orf43 was highly expressed in the regenerated liver. We hypothesized that this might be related to hepatocyte proliferation and liver regeneration.

For our work on the role of C3orf43 in normal hepatocytes, BRL3A rat liver cells were used as models for MTT and flow cytometry analysis. The results of MTT array indicated that C3orf43 significantly promoted BRL-3A cell viability. Considering the evidence that the percentage of S-phase and G2/M-phase cells significantly increased in our pCDH-C3 group compared to the pCDH group, we concluded that upregulation of C3orf43 by pCDH-C3 significantly accelerated hepatocyte proliferation, while downregulation of C3orf43 by C3-siRNA reduced hepatocyte proliferation.

To further study the molecular mechanisms of C3orf43 on proliferation in BRL-3A cells, we used RT-PCR and western blot to measure the expression of the cell proliferation-related genes JUN, MYC, CCND1 and CCNA2 in the C3-siRNA and negative control group and the pCDH-C3 and pCDH group. We also found that upregulation of C3orf43 by pCDH-C3 increased the expression of JUN, MYC, CCND1 and CCNA2, while downregulation of C3orf43 by C3-siRNA inhibited their expression.

It has been reported that c-JUN and c-MYC are immediate early genes in liver regeneration, inducing the transition of liver cells from the G0 phase to G1 phase, regulating the start-up phase of the cell cycle, and initiating the liver regeneration process [19–21]. Previous study revealed that, as an important regulatory element of liver cell proliferation and survival, c-JUN plays an important role in the process of liver regeneration [22–24].

As an oncogene, c-MYC could promote cell proliferation. It was found that increased expression of c-MYC is related to cyclin D1 and cyclin E, while c-MYC silencing reduces the expression of cyclin D1 and cyclin E and delays the function of epithelial repair [25–27]. Recent studies have reported that c-MYC is an important driver of replication in the two most commonly employed rat β-cell lines [28]. Cyclin D1 is not only an important sign to detect liver cell transformation to the G1 phase, but also an important target protein with a variety of mitogen [23, 29, 30]. Previous study showed that activator protein 1 (AP-1) has some binding sites in the promoter region of cyclin D1, and a combination of c-FOS or c-JUN would induce the expression of cyclin D1 at the mRNA level [31]. Some recent studies have shown that cyclin D1 is obviously upregulated in liver cells after partial hepatectomy, and that it regulates cells through the G1-phase checkpoint and promotes cell proliferation [32, 33].

Our data suggest that the C3-siRNA group downregulates the expression of JUN, MYC, CCND1 and CCNA2 at mRNA and protein levels, while the pCDH-C3 group upregulates their expression. Taken together, we inferred that C3orf43 may activate JUN, MYC, CCND1 and CCNA2 to promote cell proliferation in rat BRL-3A liver cell.

## Conclusions

Our results show that C3orf43 was rapidly induced at the proliferation phase of liver regeneration. C3orf43 promoted hepatocyte proliferation and cell growth by modulating the expression of the cell proliferation-related genes JUN, MYC, CCND1 and CCNA2. Our findings reveal that C3orf43 could serve as a promoter of hepatocyte proliferation, and may have the potential to promote liver repair and regeneration. In the future, we will investigate the mechanism of C3orf43 in the regulation of cell proliferation in vitro and in vivo.

## Abbreviations

C3-siRNA: the interference group with siRNA of C3orf43
DMSO: dimethylsulfoxide
MTT: methyl thiazolyl tetrazolium
pCDH: the empty vector of the overexpression group
pCDH-C3: the overexpression group with pCDH-C3orf43
PI: propidium iodide

## References

[1] Michalopoulos GK1, DeFrances MC (1997). Liver regeneration. Science.

[2] Fausto N (2000). Liver regeneration. J Hepatol.

[3] Taub R (2004). Liver regeneration: from myth to mechanism. Nat Rev Mol Cell Biol.

[4] Fausto N, Campbell JS, Riehle KJ (2006). Liver regeneration. Hepatology.

[5] Xu D, Yang F, Yuan JH, Zhang L, Bi HS, Zhou CC, Liu F, Wang F, Sun SH (2013). Long noncoding RNAs associated with liver regeneration 1 accelerates hepatocyte proliferation during liver regeneration by activating Wnt/betacatenin signaling. Hepatology.

[6] Gerhard DS, Wagner L, Feingold EA, Shenmen CM, Grouse LH, Schuler G (2004). The status, quality, and expansion of the NIH full-length cDNA project: the mammalian gene collection (MGC). Genome Res.

[7] Strausberg RL, Feingold EA, Grouse LH, Derge JG, Klausner RD, Collins FS (2002). Generation and initial analysis of more than 15,000 full-length human and mouse cDNA sequence. Proc Natl Acad Sci U S A.

[8] Geng X, Wei H, Shang H, Zhou M, Chen B, Zhang F, Zang X, Li P, Sun J, Che J, Zhang Y, Xu C (2015). Proteomic analysis of the skin of Chinese giant salamander (Andriasdavidianus). J Proteome.

[9] Dai K, Chen R, Ding Y, Niu Z, Fan J, Xu C (2014). Induction of functional hepatocyte-like cells by overexpression of FOXA3 and HNF4alpha in rat bone marrow Mesenchymal stem cells. Cells Tissues Organs.

[10] Ding Y, Chang C, Niu Z, Dai K, Geng X, Li D, Guo J, Xu C (2016). Overexpression of transcription factor Foxa2 and Hnf1α induced rat bone mesenchymal stem cells into hepatocytes. Cytotechnology.

[11] Koniaris LG, McKillop IH, Schwartz SI, Zimmers TA (2003). Liver regeneration. J Am Coll Surg.

[12] Jiao H, Zhu Y, Lu S, Zheng Y, Chen H (2015). An integrated approach for the identification of HNF4alpha-centered transcriptional regulatory networks during early liver regeneration. Cell Physiol Biochem.

[13] Mao SA, Glorioso JM, Nyberg SL (2014). Liver regeneration. Transl Res.

[14] Fitz Gerald MJ, Webber EM, Donovan JR, Fausto N (1995). Rapid DNA binding by nuclear factor kappa B in hepatocytes at the start of liver regeneration. Cell Growth Differ.

[15] Cressman DE, Diamond RH, Taub R (1995). Rapid activation of the Stat3 transcription complex in liver regeneration. Hepatology.

[16] Michalopoulos GK, Khan Z (2005). Liver regeneration, growth factors, and amphiregulin. Gastroenterology.

[17] Huh CG, Factor VM, Sánchez A, Uchida K, Conner EA, Thorgeirsson SS (2004). Hepatocyte growth factor/c-met signaling pathway is required for efficient liver regeneration and repair. Proc Natl Acad Sci U S A.

[18] Borowiak M, Garratt AN, Wüstefeld T, Strehle M, Trautwein C, Birchmeier C (2004). Met provides essential signals for liver regeneration. Proc Natl Acad Sci U S A.

[19] Alcorn JA, Feitelberg SP, Brenner DA (1990). Transient induction of c-jun during hepatic regeneration. Hepatology.

[20] Morello D, Lavenu A, Babinet C (1990). Differential regulation and expression of jun, c-fos and c-myc proto-oncogenes during mouseliver regeneration and after inhibition of protein synthesis. Oncogene.

[21] Miquet JG, Freund T, Martinez CS, González L, Díaz ME, Micucci GP, Zotta E, Boparai RK, Bartke A, Turyn D, Sotelo AI (2013). Hepatocellular alterations and dysregulation of oncogenic pathways in the liver of transgenic mice overexpressing growth hormone. Cell Cycle.

[22] Behrens A, Sibilia M, David JP, Möhle-Steinlein U, Tronche F, Schütz G, Wagner EF (2002). Impaired postnatal hepatocyte proliferation and liver regeneration in mice lacking c-jun in the liver. EMBO J.

[23] Chen YX, Zhang XR, Xie WF, Li S (2004). Effects of taurine on proliferation and apoptosis of hepatic stellate cells in vitro. Hepatobiliary Pancreat Dis Int.

[24] Ledda-Columbano GM, Pibiri M, Molotzu F, Cossu C, Sanna L, Simbula G, Perra A, Columbano A (2004). Induction of hepatocyte proliferation by retinoic acid. Carcinogenesis.

[25] Kinney EL, Tanida S, Rodrigue AA, Johnson JK, Tompkins VS, Sakamuro D (2008). Adenovirus E1A oncoprotein liberates c-Myc activity to promote cell proliferation through abating Bin1 expression via an Rb/E2F1-dependent mechanism. J Cell Physiol.

[26] Rohan JN, Weigel NL (2009). 1Alpha, 25-Dihydroxyvitamin D3 reduces c-Myc expression, inhibiting proliferation and causing G1 accumulation in C4-2 prostate cancer cells. Endocrinology.

[27] Liu L, Rao JN, Zou T, Xiao L, Smith A, Zhuang R, Turner DJ, Wang JY (2012). Activation of Wnt3a signaling stimulates intestinal epithelial repair by promoting c-Myc-regulated gene expression. Am J Physiol Cell Physiol.

[28] Karslioglu E, Kleinberger JW, Salim FG, Cox AE, Takane KK, Scott DK, Stewart AF (2011). cMyc is a principal upstream driver of beta-cell proliferation in rat insulinoma cell lines and is an effective mediator of human beta-cell replication. Mol Endocrinol.

[29] Haga S, Ogawa W, Inoue H, Terui K, Ogino T, Igarashi R, Takeda K, Akira S, Enosawa S, Furukawa H, Todo S, Ozaki M (2005). Compensatory recovery of liver mass by Akt-mediated hepatocellular hypertrophy in liver-specific STAT3-deficient mice. J Hepatol.

[30] Riehle KJ, Campbell JS, McMahan RS, Johnson MM, Beyer RP, Bammler TK, Fausto N (2008). Regulation of liver regeneration and hepatocarcinogenesis by suppressor of cytokine signaling 3. J Exp Med.

[31] Albanese C, Johnson J, Watanabe G, Eklund N, Vu D, Arnold A, Pestell RG (1995). Transforming p21ras mutants and c-Ets-2 activate the cyclin D1 promoter throughdistinguishable regions. J Biol Chem.

[32] Nelsen CJ, Rickheim DG, Timchenko NA, Stanley MW, Albrecht JH (2001). Transient expression of cyclin D1 is sufficient to promote hepatocyte replication and liver growth in vivo. Cancer Res.

[33] Rickheim DG, Nelsen CJ, Fassett JT, Timchenko NA, Hansen LK, Albrecht JH (2002). Differential regulation of cyclins D1 and D3 in hepatocyte proliferation. Hepatology.

